# Live bird market in Bangladesh: Regulatory systems and operations

**DOI:** 10.5455/javar.2021.h559

**Published:** 2021-11-06

**Authors:** Nusrat Irin, Syeda Munira Dilshad, Abdullah Al Sattar, Nurun Nahar Chisty, Afsana Sultana, Mahmudul Hasan, Rashed Mahmud, Syed Shahid Abbas, Guillaume Fournie, Md. Ahasanul Hoque

**Affiliations:** 1 Department of Medicine and Surgery, Chattogram Veterinary and Animal Sciences University, Chattogram, Bangladesh; 2 Animal Health Research Division, Bangladesh Livestock Research Institute, Dhaka, Bangladesh; 3 Institute of Development Studies, University of Sussex Falmer, Brighton, UK; 4 Department of Pathobiology and Population Sciences, The Royal Veterinary College, University of London, UK

**Keywords:** Live bird market, policy, regulation, stakeholders

## Abstract

**Objective:**

In developing countries, such as Bangladesh, the live bird market (LBM) is a vital location for the trading of live poultry. The study was carried out in nine LBMs located around Bangladesh to ascertain the present regulations and procedures governing their operation. Additionally, the responsibilities and levels of engagement of the stakeholders were determined.

**Materials and Methods:**

The data were gathered through the use of a semi-structured interview guide. Thematic analysis was used to code the interview transcripts iteratively.

**Results:**

The findings indicated that the government was directly and indirectly involved in the leasing process of the markets. A market in this country is divided into numerous sectors, including LBM, fish market, vegetable market, and grocery stores. A market’s hygienic condition is highly dependent on market authority’s decisions. In some markets, market officials conducted routine sanitary inspections. Veterinarians played a little role in the inspection procedure.

**Conclusion:**

There is no adequate, functional monitoring system to ensure that LBMs adhere to cleanliness and adequate and functional biosecurity. Biosecurity enhancements, effective cleaning programs, and regular monitoring by relevant authorities are critical for LBMs in Bangladesh.

## Introduction

The final node in the poultry production and distribution network (PDN) is the live bird market (LBM). Diverse PDNs collaborate to supply live birds, meat, and eggs for human consumption [1]. Most of the poultry in Bangladesh is marketed through LBMs [2]. These LBMs serve as important points of interaction between humans and live chickens, making them critical sources of viral infection [3,4]. Low biosecurity requirements [5] are also thought to play a role in the establishment and spread of zoonotic diseases to humans [6–8]. Indeed, zoonotic pathogens such as avian influenza viruses (AIV), *Campylobacter*, and *Salmonella* have been detected in Bangladeshi LBMs frequently [9–13]. *Salmonella* infection can occur during the preparation of poultry carcasses or in close contact with live poultry [8]. Untreated water collected from tube wells or ponds is stored in open containers and used for poultry drinking water. Personal protective equipment is not used; hand washing, cleaning, and disinfection of LBM equipment and the surrounding environment are rarely practiced. These factors are almost certainly involved in the survival and spread of health hazards [14]. Monitoring the LBM properly may be a solution to this problem [15]. 

While various studies have been conducted in Bangladesh to investigate the health concerns associated with LBMs [4,7,10], there is a dearth of knowledge regarding the regulatory environment and problems confronting LBMs. As a result, this paper examines Bangladesh’s existing policies and regulations governing LBMs, as well as their implementation. Additionally, the parties engaged in operating LBMs were identified, as were their roles in their governance. The outcomes of this study will aid in our understanding of LBM governance and will assist in the establishment of new regulations that can be effectively implemented.

## Materials and Methods

### Ethical approval

The study was conducted under the following research approval no: CVASU/Dir (R&E) EC/2020/165 (2), dated: 09/03/2020. We obtained oral consent from each participant before the interview.

### Data collection

**Figure 1.**
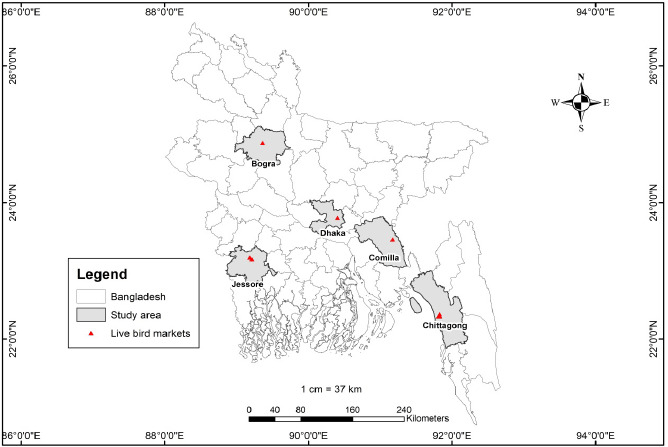
Spatial distribution of nine LBMs across Bangladesh [17,18].

From 18 to 29 November 2020, we carried out a study to understand better the regulatory processes and operations of LBMs in Bangladesh. We chose nine LBMs at random from five districts: Chattogram (four), Dhaka (one), Cumilla (one), Jashore (two), and Bogura (one) (Fig. 1). Although the Hat–Bazar rules and regulations [16] apply to all markets (including LBMs) in Bangladesh, its implementation may vary according to geographical locations, market size, ownership, and management. As a result, we chose nine markets from five districts to examine the country’s commonalities and differences. 

We interviewed members of market committees (*n* = 3), market authorities or local government (*n* = 2), and vendors (*n* = 4) in each market. The market committee comprises members who are accountable for the market’s management and regulation. Each market is required to follow its own set of regulations [16]. Vendors are traders who offer their wares to market clients. They can be the stall’s owner, lease, or private tenant. These very knowledgeable and experienced stakeholders were purposefully chosen for their expertise, experience, and impression. The data were gathered using a semi-structured pre-developed interview guide. We scheduled meetings with study participants. Face-to-face interviews were carried out, with some follow-up information acquired via phone where necessary. Each interview lasted between 20 and 25 min. While the interview guide was written in English, local speakers did the interviews themselves in Bengali. The following topics were investigated: market ownership, leasing, and taxation, market hygiene and waste management, and the technical person’s role in the market. Physical observations of the LBMs were also made to elicit information about the market environment and actor behavior.

### Data analysis

Transcripts of the raw data were created. For accuracy, transcripts were read and re-read. The thematic analysis provides a versatile framework for organizing qualitative data, identifying patterns, and conducting interpretations. We interpreted the data using theme analysis [19]. Each transcript was assigned an initial code. The primary author then reviewed the codes in order to develop themes. Prior to finishing the codes and themes, a senior researcher reviewed them.

## Results

We found that several actors were involved in running the markets, namely i) government (city corporation, district council, Upazilla Porishod and municipality); ii) market committee; iii) leaseholders; iv) private owners; v) stall owners; vi) food inspector; vii) veterinarian; viii) cleaner; and ix) consumers. 

Three broad themes (Supplementary Tables 1 and 2) were identified in relation to the objectives of the study. They were:

i) Administration and legislation system of LBMs; 

ii) Hygiene and waste management system;

iii) Inspection and supervision of LBMs.

Each of the themes is described below.

### Administration and legislation system of LBMs

Two of the nine selected LBMs were privately owned, namely the Karnafuli Complex in Chattogram and the Wireless Kacha Bazar in Dhaka. Each LBM shall adhere to the legislative system’s government Hat–Bazar norms [16]. The local government directly administered only one market (Jashore municipality). The city corporation or municipality held the remaining seven markets. The city corporation or municipality leased the remaining six markets to a market committee.

Lease terms and duration varied according to the market. Leases can be renewed annually, 3/5 years, or for longer periods of time. Leaseholders may be the market committee, individual stallholders (grouped together), or a single authority (who are not involved in any market committee or a group). The leaseholder is chosen through a competitive process. The leaseholder is selected based on the highest bid. The city corporation/municipality publishes the tender notice and specifies the deadline for submissions. A lease’s baseline amount was determined by the average amount collected from the market over the preceding 3 years. To apply for a lease, applicants must purchase a “schedule.” The schedule’s price is determined by the lead’s price. 30% of the lease should be paid as a deposit—25% of the lease should be paid as a guarantee—and 5% should be paid as a guarantee. Following receipt of applications from qualified candidates, the municipality/city corporation convenes a tender committee meeting to assess the bids. As previously stated, the municipality/city corporation rents the market to the highest bidder. The lease is required to pay the remaining 75% in installments.

Markets adhere to government tax regulations. Vendors rent stalls and pay daily rent to the market leaseholder. Rent is determined by the size of the stall and its location within the market.

### Hygiene and waste management system

We discovered some intriguing differences in the manner in which markets conduct cleaning and hygiene programs. Only the local government (i.e., city corporation or municipality) or leaseholders are responsible for cleaning five out of nine markets. The local government employs a small number of people who clean the market once or twice daily. They clean the market with detergent, soap, and other household cleaners. Stall owners were responsible for cleaning and disinfecting their markets around twice/thrice a week in each of the three markets. However, the cleaning process and frequency are determined by the vendors. At one market, the Chittagong Development Authority (CDA) Karnafuli market in Chattogram, both the market authority (a private owner) and stall owners are responsible for maintaining the market’s cleanliness. Generally, waste is deposited in a receptacle outside the marketplace and collected daily by city corporation staff.

Markets where the authority does not monitor or supervise management and cleaning activities regularly appeared to be dirtier than markets where the authority, or both the authority and sellers, participates in cleaning efforts. For example, sellers in Chattogram’s Reazuddin Bazar and Dewanhat market cleaned their markets once or twice a week; cages were filthy and overflowing with trash debris.

We included an additional question to get further information about the participant’s LBMs. We discovered several difficulties as a result of this. The sellers at Chattogram’s CDA Karnafully market complained about a lack of water supply and advised installing deep tube wells for improved cleaning. On the other hand, stall owners in Cumilla and Bogura urged extending the space. The traders in Wireless Kancha Bazar in Dhaka stated that they suffered a significant loss during coronavirus disease 2019. As a result, nearly two-thirds of live bird sellers ceased operations. In Jashore, the slaughtering area’s hygiene was poor and required improvement.

### Inspection and supervision of LBMs

Five of the nine LBMs carried out routine checks. The market authority assigned an officer. This inspection served a variety of functions, including ensuring adequate hygiene, food safety, and the pricing of various commodities. These inspections did not take place at a predetermined time. It occurred on average once or twice a week. The markets where the examination took place appeared to be more orderly and tidy than other markets. Although this was not part of the inspection procedure, a veterinarian took biological samples from LBMs in three markets.

A surprise mobile court operation was conducted in the market to investigate commodity prices and quality, consumer rights, hygiene, and management systems. At the time this article was written, it was discovered that two markets did not have mobile court operations. However, mobile court operations had occurred in the remaining seven markets. Experts guide the court in investigating illegal and detrimental market activity and taking effective action against them. Typically, the mobile court includes representatives from the city corporation, district administration, and law and enforcement agency, as well as a technical expert from another government organization (Department of Livestock Services, Fisheries, and Environment), as well as representatives from the Consumers Association of Bangladesh and the market committee [20].

## Discussion

The leasing mechanism was one of the most distinguishing characteristics of the evaluated LBMs. The local government (city corporation and municipality) has a direct or indirect stake in the market through the lease. The lease is awarded to the candidate or group with the highest financial capacity. Additionally, the leaseholder is responsible for the markets’ administrative and hygienic maintenance. It is critical to assess the leaseholders’ regulatory capacity and potential to ensure consistent management of the LBMs. The inspection system contributes to the preservation of market hygiene and biosecurity. The engagement of all market participants is necessary for the LBMs to operate. Who controls the market, what they do, and other affiliated partners all contribute to the market’s environment.

It was shown that almost all LBMs lacked adequate hygiene and sanitation, posing a risk of disease transmission. The birds were overcrowded, creating ideal conditions for multiplying and sustaining virus circulation, and so posing the risk of becoming viral reservoirs themselves [1]. Due to the low cleaning frequency of LBMs, they may operate as drivers of viral evolution, favoring the generation of novel variations [5]. Consumption of meat from these markets is becoming more well recognized as a possible cause of Salmonellosis [10]. Physical assessment of the market revealed two marketplaces with inadequate hygiene in the slaughter area; this could be a source of *Salmonella* infection for the human population [8]. *Salmonella* sp. contamination of chicken workers indicated a possible failure of personal hygiene during the handling and processing of chickens [7]. The majority (50%–80%) of human cases of Campylobacteriosis are caused by the eating of chicken products [21]. Overcrowding and a constant supply of susceptible birds of various types and breeds may foster the silent transmission of AIVs in these marketplaces [11,22]. In the LBMs, the poultry-to-poultry transmission of avian influenza is widespread. Birds entering the LBM system via wholesale markets may be susceptible to influenza infection due to crowded conditions, travel stress, and housing in a contaminated environment [23]. On LBM, hygiene is required because infected live birds harbor and spread *Salmonella* to other birds by lateral transmission, primarily via feces, soil, litter, feeds, water, dust, and feathers [24].

According to the study, an effective surveillance system and appropriate regulation of the live bird industry will minimize disease persistence and transmission. The market’s competent authority must ensure that the market is administered effectively. This paper analyzed nine LBMs in Bangladesh, which appears insufficient to acquire a comprehensive picture of the regulatory structure and administration. It has, however, uncovered numerous exciting ideas that will act as a springboard for further inquiry.

The study encompassed nine local Live Bird Markets (LBMs) in five districts, with Chattogram alone covering four markets (Table 1). Due to time and resource constraints, the remaining places included only one or two markets. As a result, the sample size was insufficient to form a conclusive idea. However, markets with varying features included in the study aided in determining the involvement of various stakeholders and demonstrating the market’s regulatory framework.

**Table 1.** Percentage and frequency for legislation system of market (*n* = 9).

Rules/documents	Percentage	Frequency
Only Government Hat–Bazar rules	66.7	6
Written private document as well as Government Hat–Bazar rules	33.3	3
Lease system		
City corporation/municipality provides lease to market committee/individual authority	66.7	6
Take lease from Bangladesh Railway Authority for 99 years	11.1	1
Private owner provides lease to the market committee	11.1	1
City corp/municipality directly controls the market	11.1	1

*n* = Number of LBMs.

## Conclusion

LBMs are the heart of the poultry trading industry, bringing together numerous merchants from various places to sell birds. Nonetheless, risks of disease introduction and strain evolution continue to rise as a result of insufficient oversight by policymakers, inspectors, and hygiene and sanitation. As a result, to conduct a complete study of the current situation, we need to obtain further information about the operation of LBMs in various areas of Bangladesh. Additionally, each stakeholder’s precise duties must be examined to create a vendor-customer-friendly policy structure and provide a consistent operating procedure in the LBMs.

- For instance, we can investigate the aspects of linked stakeholders’ views, attitudes, and perceptions in order to ascertain their demands and responsibilities.
- The variables contribute to the formation of a specific attitude or perception, as well as their decision-making processes.
- The difficulties they encountered in their service and operation systems, as well as strategies for improving the system and developing a sound policy.

## List of abbreviations

AIV, Avian influenza virus; CDA, Chittagong Development Authority; LBM, Live bird market; PDN, Poultry Production and Distribution Network.

## Figures and Tables

**Supplementary Table 1.** Frequency and percentage of the market under theme (*n* = 9).

Theme	Sub-theme	Code	Frequency	Percentage
1. Administration and legislation system of LBMs	1.1. Authority/ownership	Private ownership	2	22.22
		Government ownership (city corporation/municipality/others)	7	77.78
	1.1. Rules/documents	Written private document as well as government Hat–Bazar rules	3	33.33
		Only government Hat–Bazar rules	6	66.67
	1.2. Lease system	City corp./municipality provides lease to market committee/individual authority	6	66.67
		Take lease from Bangladesh Railway Authority for 99 years	1	11.11
		Private owner provides lease to market committee	1	11.11
		City corp/municipality directly controls the market	1	11.11
	1.3. Taxation and rent system	Depends on stall holders income followed by govt. rules	9	100
		Pay a daily rent to the leaseholder/market committee based on stall size and others	9	100
2. Hygiene and waste management program/function	2.1. Hygiene and cleaning	Only authority (city cop/municipality/market committee/ private owner/leaseholder)is responsible for cleaning the market	5	55.56
		Only stall owners in a group are responsible for cleaning the market	3	33.33
		Both authority and stall owners, respectively, clean the market	1	11.11
	2.2. Waste management	City corporation/municipality collects the wastage	9	100
		Use dustbin that is located outside of the market	9	100
3. Inspection and supervision of LBMS	3.1. Inspection system	Authority (city crop/municipality/owner) sends market inspector in regular basis	5	55.56
		No inspection system	4	44.44
	3.2. Technical person involvement (vet)	Local livestock and fisheries officer visits sometimes (but not as a part of inspection team	3	33.33
		No involvement/visit of vet	6	66.67
	3.3. Mobile court	Frequent mobile court operation	7	77.78
		No mobile court operation occurs till now	2	22.22
*n* = Number of LBMs.				

**Supplementary Table 2.** Distribution of the markets according to sub-theme and coding (*n* = 9).

Sub-theme	Code	Chattogram LBMs				Dhaka	Cumilla	Bagura	Jashore		
		CDA Karnafuli	Karnafuli complex	Reazuddin bazar	Dewanhat bazar	Wireless Kacha bazar	Ranir bazar	Uposhohor bazar	Boro bazar	Hat–bazar	Frequency
Authority	Private ownership		1			1					2
	Government ownership (city corporation/pauroshova/others)	1		1	1		1	1	1	1	7
Total											9
Rules/documents	Written private document as well as government Hat–Bazar rules	1	1				1				3
	Only government Hat–Bazar rules			1	1	1		1	1	1	6
Total											9
Lease system	City corp./municipality provides lease to market committee/individual authority/any group	1		1	1		1	1	1		6
	Take lease from Bangladesh Railway Authority for 99 years		1								1
	Private owner provides lease to market committee					1					1
	City corp/municipality directly controls the market									1	1
Total											9
Taxation and rent system	Depends on stallholders income followed by govt. rules	1	1	1	1	1	1	1	1	1	9
	Pay a daily rent to the leaseholder/market committee based on stall size and others	1	1	1	1	1	1	1	1	1	9
Total											
Hygiene and cleaning	Only authority (city cop/municipality/market committee/ private owner/leaseholder) is responsible for cleaning the market		1			1	1		1	1	5
	Only stall owners in a group are responsible for cleaning the market			1	1			1			3
	Both authority and stall owners, respectively, clean the market	1									1
Total											9
Waste management	City corporation/municipality collects the wastage	1	1	1	1	1	1	1	1	1	9
	Use dustbin that is located far outside of the market (definition of far-needs to be defined)	1	1					1			3
	Use dustbin that is located near to the market (definition of near-needs to be defined)			1	1	1	1		1	1	6
Total											9
Inspection system	Authority (city crop/municipality/owner) sends market inspector in regular basis	1	1				1		1	1	5
	No inspection system			1	1	1		1			4
Total											9
Technical person involvement (vet)	Local livestock and fisheries officer visits sometimes (but not as a part of inspection team)	1					1	1			3
	No involvement/visit of vet		1	1	1	1			1	1	6
Total											9
Mobile court	Frequent mobile court operation	1	1	1	1		1		1	1	7
	No mobile court operation occurs till now					1		1			2
Total											9

LBM = Live bird market.

*n* = Number of LBMs.
